# A cross-sectional study of the endorsement proportion of reporting guidelines in 1039 Chinese medical journals

**DOI:** 10.1186/s12874-022-01789-1

**Published:** 2023-01-21

**Authors:** Yuting Duan, Lingyun Zhao, Yanfang Ma, Jingyuan Luo, Juexuan Chen, Jiangxia Miao, Xuan Zhang, David Moher, Zhaoxiang Bian

**Affiliations:** 1 Hong Kong Chinese Medicine Clinical Study Center, School of Chinese Medicine, Hong Kong Baptist University, Hong Kong SAR, China; 2 Chinese EQUATOR Center, Hong Kong Baptist University, 3/F, Jockey Club School of Chinese Medicine Building, 7 Baptist University Road, Kowloon Tong, Hong Kong SAR, China; 3 Evidence-based Research Office, The Affiliated TCM Hospital of Guangzhou Medical University, Guangzhou, China; 4 Centre for Chinese Herbal Medicine Drug Development, Hong Kong Baptist University, Hong Kong SAR, China; 5 Pediatric TCM Clinic, Guangzhou Women and Children’s Medical Center, Guangzhou, China; 6 School of Chinese Medicine, The Chinese University of Hong Kong, Hong Kong SAR, China; 7 Canadian EQUATOR Centre, The Ottawa Hospital, General Campus, Centre for Practice Changing Research Building, 501 Smyth Road, PO BOX 201B, Ottawa, ON K1H 8L6 Canada; 8 Ottawa Methods Center, Ottawa Hospital Research Institute, The Ottawa Hospital, Ottawa, Canada

**Keywords:** Reporting guideline, CONSORT, Clinical study, Reporting quality, Transparency

## Abstract

**Background:**

Reporting quality is a critical issue in health sciences. Adopting the reporting guidelines has been approved to be an effective way of enhancing the reporting quality and transparency of clinical research. In 2012, we found that only 7 (7/1221, 0.6%) journals adopted the Consolidated Standards of Reporting Trials (CONSORT) statement in China. The aim of the study was to know the implementation status of CONSORT and other reporting guidelines about clinical studies in China.

**Methods:**

A cross-sectional bibliometric study was conducted. Eight medical databases were systematically searched, and 1039 medical journals published in mainland China, Hong Kong, Macau, and Taiwan were included. The basic characteristics, including subject, language, publication place, journal-indexed databases, and journal impact factors were extracted. The endorsement of reporting guidelines was assessed by a modified 5-level evaluation tool, namely i) positive active, ii) positive weak, iii) passive moderate, iv) passive weak and v) none.

**Results:**

Among included journals, 24.1% endorsed CONSORT, and 0.8% endorsed CONSORT extensions. For STROBE (STrengthening the Reporting of Observational Studies in Epidemiology), PRISMA (Preferred Reporting Items for Systematic Reviews and Meta-Analyses), STARD (An Updated List of Essential Items for Reporting Diagnostic Accuracy Studies), CARE (CAse REport guidelines), the endorsement proportion were 17.2, 16.6, 16.4, and 14.8% respectively. The endorsement proportion for SPIRIT (Standard Protocol Items: Recommendations for Interventional Trials), TRIPOD (Transparent Reporting of a Multivariable Prediction Model for Individual Prognosis or Diagnosis), AGREE (Appraisal of Guidelines, Research, and Evaluation), and RIGHT (Reporting Items for Practice Guidelines in Healthcare) were below 0.7%.

**Conclusions:**

Our results showed that the implementation of reporting guidelines was low. We suggest the following initiatives including i) enhancing the level of journal endorsement for reporting guidelines; ii) strengthening the collaboration among authors, reviewers, editors, and other stakeholders; iii) providing training courses for stakeholders; iv) establishing bases for reporting guidelines network in China; v) adopting the endorsement of reporting guidelines in the policies of the China Periodicals Association (CPA); vi) promoting Chinese medical journals into the international evaluation system and publish in English.

**Supplementary Information:**

The online version contains supplementary material available at 10.1186/s12874-022-01789-1.

## Introduction

Quality and transparency are essential for clinical research, which can promote the results of the clinical trials as clinical evidence, thus affecting the decision-making of clinical practice. Reporting guidelines have been proven to be useful tools for enhancing the quality and transparency of clinical research [1]. Since the Consolidated Standards of Reporting Trials (CONSORT) statement was first published in 1996 [2], other reporting guidelines for clinical studies and their secondary studies, such as Preferred Reporting Items for Systematic Reviews and Meta-Analyses (PRISMA) [3], STrengthening the Reporting of Observational Studies in Epidemiology (STROBE) [4], Standard Protocol Items: Recommendations for Interventional Trials (SPIRIT) [5], An Updated List of Essential Items for Reporting Diagnostic Accuracy Studies (STARD) [6], Transparent Reporting of a Multivariable Prediction Model for Individual Prognosis or Diagnosis (TRIPOD) [7], CAse REport guidelines (CARE) [8], Appraisal of Guidelines, Research, and Evaluation (AGREE) [9] and Reporting Items for Practice Guidelines in Healthcare (RIGHT) [10] were developed over the last 25 years. Reporting guidelines provide researchers, peer-reviewers, editors, and other stakeholders a simple and feasible method to assess whether authors have reported on these items, which are the minimum set for the basic information of clinical research. If reporting guidelines can be adequately followed, the reporting quality of research will be effectively improved, the methodological quality of research will be easily evaluated, and the transform of the research results will be accelerated realized [11].

China makes a large contribution to clinical research. The number of Chinese clinical medicine research papers reached 44,279 in 2018 and ranked second in the world [12]. During the past 10 years, the biomedical research community is witnessing a proliferation of clinical research from China [13, 14]. Evidence-based medicine worldwide needs clinical research evidence from China [15, 16]. During the process of promoting Chinese study evidence to the world, the quality, and transparency of clinical research in China are crucial. Medical journals are acting as a gatekeeper for the dissemination of the research findings. The results of Chinese medical studies published in international peer-review medical journals only account for a small part and the vast majority of them are still published in Chinese medical journals [15].

We found that only 7 Chinese medical journals adopted the CONSORT statement in 2012 [17]. In the previous study, we only searched the China Academic Journals (CAJ) Full Database which included the main medical journals in mainland China. The aim of the study is to know the current status of reporting guidelines endorsement in Chinese medical journals.

## Methods

A cross-sectional study was conducted. The Strengthening the Reporting of Observational Studies in Epidemiology (STROBE) for cross-sectional checklist was followed.

### Inclusion criteria

All medical journals that published clinical studies, systematic reviews/meta-analyses, and clinical practice guidelines, which were published in China including Mainland China, Hong Kong, Macau, and Taiwan were included. There was no language of publication restrictions.

### Exclusion criteria

Journals that have ceased publication were excluded. Journals lacking official websites were also excluded.

### Identification of Chinese medical journals

The Chinese Biomedical Literature Service System (CBM) [18], China National Knowledge Infrastructure (CNKI) [19], Wanfang Data [20], and VIP Chinese Medical Journal Databases [21] were systematically searched for the medical journals from mainland China. The Hong Kong Macau Periodicals Network [22], HKInChip [23], Macau Periodical Index [24], and Airiti Library [25] were systematically searched for the medical journals from Hong Kong and Macau. The Airiti Library was used to search for medical journals from Taiwan. All medical journals in the databases and networks listed under the heading “Journal Navigation” were examined. We determined whether the journal included clinical studies, systematic reviews/meta-analyses, and clinical practice guidelines by i) the classification of journals in the databases, ii) the texts on the introduction of journals and submission guidelines for authors, and iii) whether clinical studies, systematic reviews/meta-analyses, and clinical practice guidelines were included in last year’s issues. If the journal had a searchable table of contents, we used the keywords “case report”, “case series”, “observational study”, “cohort study”, “cross-sectional study”, “case–control study”, “controlled trial”, “clinical trial”, “clinical study”, “systematic review”, “meta-analysis”, and “clinical practice guideline” to search for the publication of clinical studies. The journals were searched and screened by two researchers (LZ and JC), confirmed by a third researcher (YD) from December 2020 to January 2021.

### Extracting data

First, the basic characteristics of the eligible journals were extracted; these included the Chinese and English names of the journal, publication place, publication institution/publisher, subjects, languages, the journal impact factor (JIF), and the official website address of each journal. Subjects were classified by referring to the journal discipline navigation within the CNKI [19]. The JIF from Science Citation Index (SCI)/Science Citation Index Expanded (SCIE) through the Web of Science database [26] were extracted for the medical journals which are included in SCI/SCIE. The JIF from the journal citation reports in the Chinese Science Citation Index [27], which is currently the most complete citation database of Chinese journal articles on mainland China, was selected as complementary data.

Second, the endorsement of reporting guidelines [3–10, 28] was extracted by systematically searching the official websites of eligible journals. For example, whether the CONSORT statement and its extensions were mentioned in the instruction for authors, author guidelines, peer reviewer guidance, editorial policies, or other relevant directions for authors of a journal and its recommendation level were extracted using a standardized form. We assessed the level of reporting guideline endorsement with a modified 5-level evaluation tool, namely i) active strong, ii) active weak, iii) passive moderate, iv) passive weak, and v) none. For the evaluation tool, we added two conditions to “active weak” and “passive moderate” based on a reference [28].

A completed CONSORT [28, 29] checklist and/or a flow diagram with article submission was assessed as “active strong”. “Active weak” was assessed as the journal “encourages” or “should” reference or follow a specific guideline; priority publication if the manuscript follows a specific guideline. “Passive moderate” were assessed as adhering to “relevant” RGs; abstracts are required to follow a specific guideline. Preparing the manuscripts according to the International Committee of Medical Journal Editors (ICMJE) was assessed as “passive weak”. No mention of any reporting guidelines was assessed as “none”.

The endorsement types of other reporting guidelines, including PRISMA, STROBE, SPIRIT, STARD, TRIPOD, CARE, AGREE, and RIGHT, which are the basic reporting guidelines related to clinical studies, systematic reviews/meta-analyses, and clinical practice guidelines were also extracted according to the above evaluation criteria.

All the above information was extracted and assessed by two researchers (LZ and JC) during the period from January to August 2021. After the first extraction, 10% of records were double-checked by two researchers (YD and YM). When disagreements happened, the judgment was made by senior researchers (ZB and DM). Primary data sources (i.e., website pages) were recorded; relevant text describing guideline endorsement was extracted and coded into a standard data extraction sheet in Excel. All the original data has been submitted as an open-source data set on the Open Science Framework platform.

### Statistical analyses

All data were collected and recorded in Microsoft Office Excel (Version 2016). Basic characteristics of included journals (clinical contents, language, publication place, journals indexed databases, and journal impact factor), endorsement type of reporting guidelines, were presented using descriptive statistics such as counts (n) and percentages (%). The bar and pie charts made by Excel 2016 were used to show the results of subjects, language, and journals indexed databases. A heat map was generated using Tableau (Version 2018.3.2) to present the number of journals in different publication places. Logistic regression was used to analyze the influencing factors of reporting guidelines endorsement. The factors associated with the endorsement of reporting guidelines were analyzed by logistic regression using SPSS (Version 25.0). The endorsement types of CONSORT statement including “active strong”, “active weak”, “passive moderate”, and “passive weak” were used as a positive outcome. The endorsement type of CONSORT, “none” was considered as a negative outcome. Whether the journal is included in the SCI/SCIE database, whether the journal is for traditional Chinese medicine (TCM), and whether the publication language of the journal includes English were included as the independent variables.

## Results

**Fig. 1:**
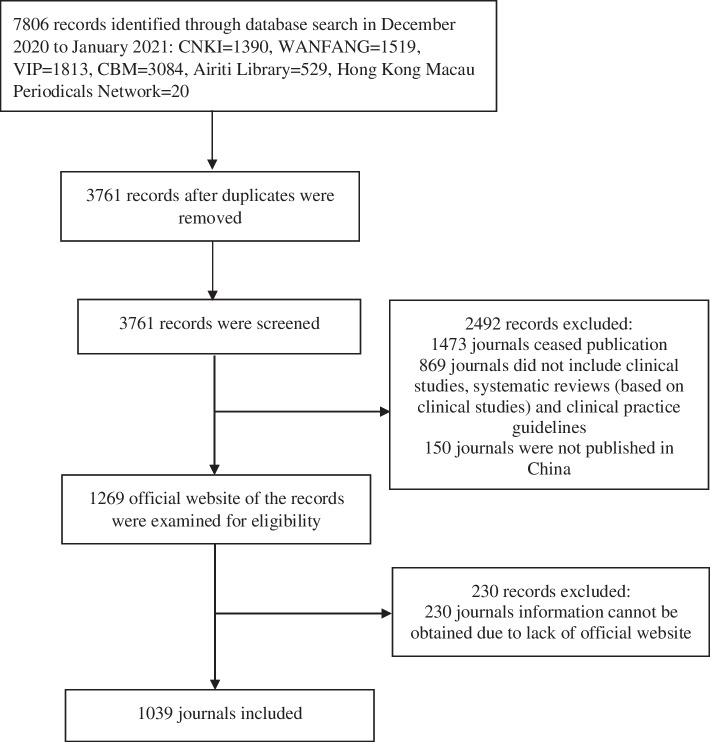
Flow chart

We initially identified 7806 journals. Of the remaining 3761 journals after removing the duplicates, 1473 had ceased publication; 869 did not include clinical studies, systematic reviews/meta-analyses, and clinical practice guidelines; 150 were not published in mainland China, Hong Kong, Macau, nor Taiwan; and 230 lacking official websites: all of these were excluded. A total of 1039 journals (Additional file 1: Appendix 1) met our inclusion criteria. Figure 1 presents the flow chart of the review process.

### Study characteristics

**Fig. 2:**
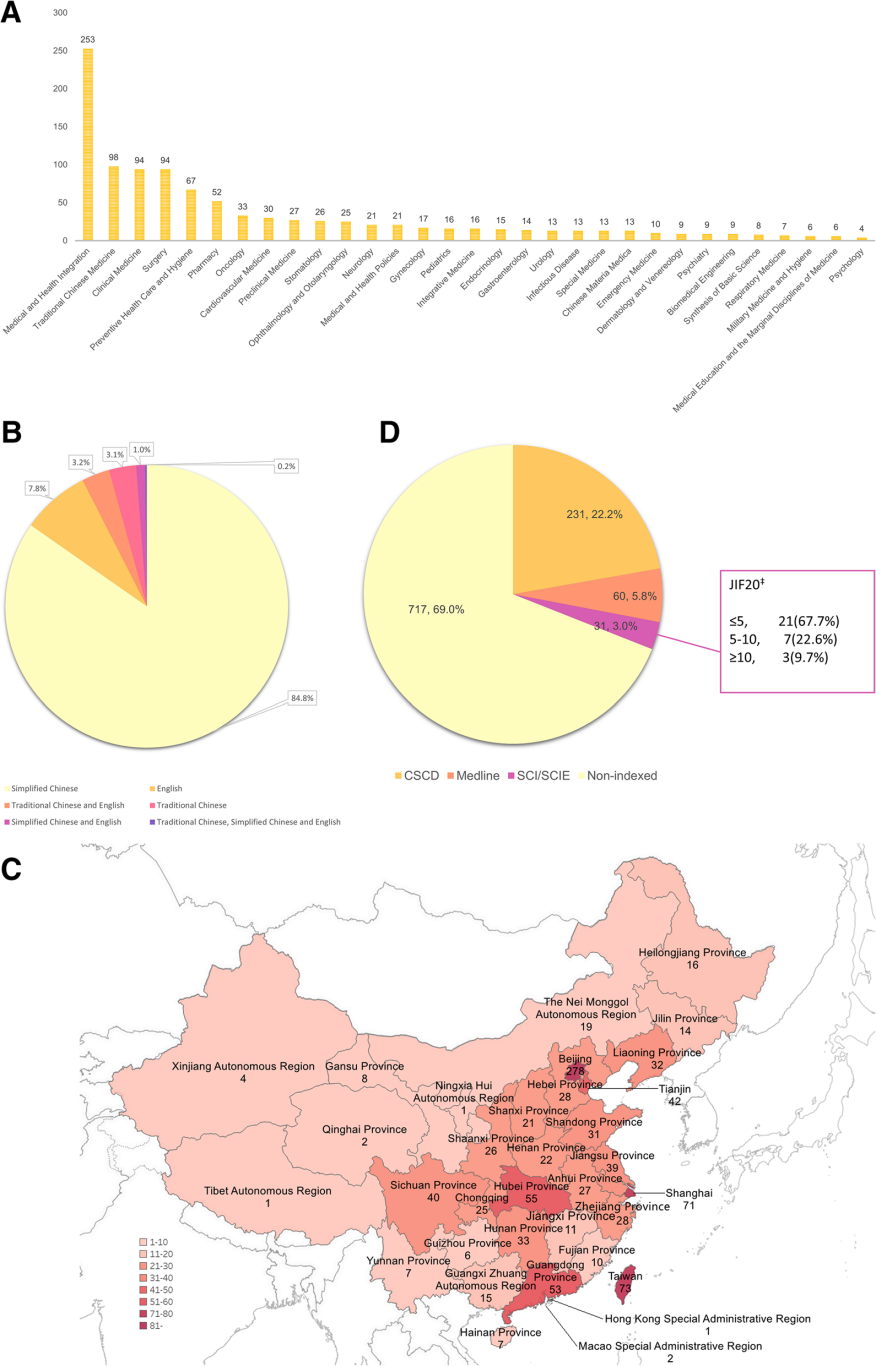
Basic characteristics of 1039 Chinese medical journals. **A** Clinical contents of 1039 Chinese medical journals. B: Publication language of 1039 Chinese medical journals. **C** Geographic distribution of journals in China. *The figure was made according to the study results using the Tableau (Version 2018.3.2). **D** Journals indexed by SCI/SCIE, Medline, and/or CSCD. ^‡^ 2020 Journal Impact Factor of SCI/SCIE

As reported in Fig. 2A, for subjects, many journals focused on Medical and Health Integration (24.4%), followed by TCM (9.4%), Clinical Medicine (9.0%), and Surgery (9.0%). For publication language (Fig. 2B), 881(84.8%) journals were published in simplified Chinese, 81 (7.8%) in English, 33 (3.2%) in traditional Chinese and English, 32 (3.1%) in traditional Chinese, 10 (1.0%) in simplified Chinese, and English, 2 (0.2%) in traditional Chinese, simplified Chinese, and English. For geographic distribution (Fig. 2C), most journals (278, 26.8%) were published in Beijing, followed by Taiwan (73, 7.0%), and Shanghai (71, 6.8%). For journals index (Fig. 2D), 231(22.2%) were indexed in SCI/SCIE, 60 (5.8%) in the International Comprehensive Biomedical Information Bibliographic Database produced by the National Library of Medicine (MEDLINE), and 31 (3.0%) in Chinese Science Citation Database (CSCD). For journals included in SCI/SCIE (Fig. 2D), only three journals (0.1%), namely Bone Research, Cellular & Molecular Immunology, and Acta Pharmaceutica Sinica B had a JIF of more than 10 in 2020. The JIFs of 21 (67.7%) journals were below 5, and 7 (22.6%) journals were between 5 to 10.

### Endorsement of reporting guidelines

The endorsement types were identified, and the examples of each endorsement type were shown in Table 1. The endorsement proportion of the CONSORT extensions and other reporting guidelines were shown in Table 2.

**Table 1:** Endorsement types defined with examples

Type	Definition	Examples
Active strong	A requirement of a completed checklist and/or a flow diagram with article submission (e.g., “must”, “should be uploaded”)	Randomized controlled trials should state whether the manuscript is written in accordance with the requirements of the CONSORT statement. For details, please refer to various CONSORT extensions and other related resources (www.consort-statement.org). (Chinese Journal of General Surgery) The article submission requires that different research designs should comply with its reporting guidelines (including the checklist). The examples include CONSORT for randomized controlled trials (www.consort-statement.org), STROBE for reporting observational studies in epidemiology (strobe-statement.org), PRISMA for meta-analysis of randomized controlled trials (prisma-statement.org), STARD for reporting diagnostic accuracy studies (www.stard-statement.org) and CARE for case reports (https://www.care-statement.org/). (Chinese Journal of Diabetes Mellitus) “Randomized controlled trials should be presented according to the CONSORT guidelines. At manuscript submission, authors must provide the CONSORT checklist accompanied by a flow diagram that illustrates the progress of patients through the trial, including recruitment, enrollment, randomization, withdrawal and completion, and a detailed description of the randomization procedure. The CONSORT checklist and template flow diagram can be found on http://www.consort-statement.org.” (The Journal of Biomedical Research)
Active weak	A suggestion that authors are “encouraged” or “should” reference or follow a specific guideline; Priority publication if follow a specific guideline	Please refer to the corresponding reporting guidelines for the format of the paper. The examples include CONSORT 2010 checklist, CONSORT-CHM Formulas checklist, Acupuncture Interventions (STRICTA) checklist, the STRICTOC checklist, COREQ for qualitative research, PRISMA checklist for reporting systematic reviews and meta-analyses. (Chinese Journal of Integrated Traditional and Western Medicine) Priority publication: Papers written in accordance with the specifications of reporting guidelines, such as the Consolidated Standards Of Reporting Trials (CONSORT) 2010 statement, CONSORT Extension for Chinese Herbal Medicine Formulas 2017. (Journal of Practical Traditional Chinese Internal Medicine) “Reporting of randomized controlled trials should follow the guidelines of The CONSORT Statement: http://www.consort-statement.org” (Chinese Journal of Cancer Research)
Passive moderate	A suggestion that authors should adhere to “relevant” reporting guidelines; Abstracts are required to follow a specific guideline	The abstract of a prospective clinical trial study should contain the essential elements listed in the CONSORT statement (http://www.consort-statement.org/home) (Chinese Journal of Digestive Surgery)
Passive weak	References documents (e.g., ICMJE or editorial policies) which mention reporting guidelines	“Shanghai Journal of Acupuncture and Moxibustion” adopts the format and requirements of the new version of “Uniform Requirements for Manuscripts Submitted to Biomedical Journals” formulated by the International Committee of Medical Journal Editors (ICMJE) (Shanghai Journal of Acupuncture and Moxibustion) “Manuscripts should be prepared in accordance with the Uniform Requirements for Manuscripts Submitted to Biomedical Journals by ICMJE (www.icmje.org)” (Journal of Acupuncture and Tuina Science)
None	No mention of any reporting guidelines	Not applicable

**Table 2:** Summary of reporting guidelines endorsement in Chinese medical journals

	**Endorsement type**				
Reporting Guideline (*n* = 1039*)	**Active strong n (%)**	**Active weak n (%)**	**Passive moderate n (%)**	**Passive weak n (%)**	**None n (%)**
CONSORT	108 (10.4)	49 (4.7)	64 (6.2)	29 (2.8)	789 (75.9)
CONSORT Extension	8 (0.8)	0 (0)	0 (0)	0 (0)	1031 (99.2)
STROBE	152 (14.6)	21 (2.0)	6 (0.6)	0 (0)	860 (82.8)
PRISMA	151 (14.5)	20 (1.9)	1 (0.1)	0 (0)	867 (83.4)
SPIRIT	2 (0.2)	5 (0.5)	0 (0)	0 (0)	1032 (99.3)
STARD	148 (14.2)	16 (1.5)	6 (0.6)	0 (0)	869 (83.6)
TRIPOD	2 (0.2)	3 (0.3)	0 (0)	0 (0)	1034 (99.5)
CARE	145 (14.0)	8 (0.8)	1 (0.1)	0 (0)	885 (85.2)
AGREE	2 (0.2)	3 (0.3)	1 (0.1)	0 (0)	1033 (99.4)
RIGHT	3 (0.3)	3 (0.3)	0 (0)	0 (0)	1033 (99.4)

*Abbreviations*: *STROBE* Strengthening the Reporting of Observational Studies in Epidemiology, *PRISMA* Preferred Reporting Items for Systematic Reviews and Meta-Analyses, *SPIRIT* Defining Standard Protocol Items for Clinical Trials, *STARD* An Updated List of Essential Items for Reporting Diagnostic Accuracy Studies, *TRIPOD* Transparent Reporting of a Multivariable Prediction Model for Individual Prognosis or Diagnosis, *CARE* Consensus-based Clinical Case Reporting Guideline Development, *AGREE* Appraisal of Guidelines, Research and Evaluation, *RIGHT* Reporting Practice Guidelines in Health Care

^*^ Number of Journals Assessed for Endorsement

Of the 1039 journals, 24% endorsed CONSORT. Among 157 (15.1%) journals that actively endorsed CONSORT, 108 (10.4%) journals required the use of CONSORT (active strong), while 49 (4.7%) journals encouraged the use of CONSORT (active weak). The endorsement of remaining journals was assessed as passive moderate and passive weak, representing 64 (6.2%) and 29 (2.8%) journals, respectively. Only 8 (0.8%) journals required the use of the CONSORT extensions.

Of the other reporting guidelines, the endorsement proportion of STROBE, PRISMA, STARD, and CARE were 17.2, 16.6, 16.4, and 14.8% respectively. Only a few journals (14.0%-14.6%) required submitting a completed checklist along with the manuscript. The remaining reporting guidelines, such as SPIRIT, TRIPOD, AGREE, and RIGHT were only mentioned in a few included journals, below 0.7%.

### Factors associated with the endorsement of reporting guidelines

Regression analysis found that i) whether the journal belongs to SCI/SCIE has an association with on the endorsement of CONSORT (OR = 3.164, 95%CI = [1.313, 7.620], *P* = 0.010); ii) whether the journal published in English has an association with the endorsement of CONSORT (OR = 1.987, 95%CI = [1.127, 3.503], *P* = 0.018); iii) there is no evidence to support whether the journal belongs to TCM has an association with the endorsement of CONSORT (OR = 0.656, 95%CI = [0.294, 1.461], *P* = 0.302). The details of the regression results are shown in Table 3.

**Table 3:** Regression analysis for influencing factors of CONSORT endorsement

**Variables**	**Groups**	**B**^‡^	**Standard Error of B**	**Wald Chi-square Value**	***P* value**	**OR**	**95%CI**
SCI	Yes No*	1.152	0.449	6.593	0.010	3.164	(1.313,7.620)
English (Publication language)	Yes No*	0.687	0.289	5.638	0.018	1.987	(1.127,3.503)
TCM	Yes No*	-0.422	0.409	1.066	0.302	0.656	(0.294,1.461)

^* ^Control group

^‡^ Partial regression coefficient

## Discussion

Our study provided a comprehensive overview of how many and to what extent Chinese medical journals adopt reporting guidelines. Taking CONSORT as an example, our previous study has shown that the number of Chinese medical journals which endorsed CONSORT consisted of less than 0.6% (7/1221) in 2012 [17]. In this study, we found that the endorsement proportion of CONSORT was 24.1% (250/1039). There is still much work that needs to be done to enhance the uptake of CONSORT and other reporting guidelines in Chinese medical journals.

The first reporting guideline CONSORT statement, which also found the development path of reporting guidelines [15]. According to CONSORT group statistics, there are currently 585 journals and over 50% of the core medical journals listed in the Abridged Index Medicus on PubMed that endorse CONSORT [29]. The CONSORT statement was first introduced to China in 2001 [30], followed by SPIRIT, PRISMA, and other reporting guidelines. After the introduction, many studies found that the positive function of reporting guidelines in improving the reporting quality of Chinese clinical research [31–36]. Meanwhile, Chinese medical journals began to endorse CONSORT and other reporting guidelines. However, according to our study results, there existed a big gap between Chinese medical journals and core medical journals in the world.

As for clinical contents of Chinese medical journals, “medical and health integration”, which is like comprehensive medical journals accounted for the largest type, followed by TCM journals. The big proportion of TCM journals reflect the feature for the medical subject in China. Therefore, the quality and transparency of TCM research can represent an important part of the level of Chinese clinical research. Since 2001, the reporting guidelines system of TCM has gradually been established [37]. The existing TCM reporting guidelines have included major study designs and main TCM interventions [38]. Although the results of this study do not show that journal endorsement in TCM is better than in other fields, given the efforts of the Chinese scholars in TCM reporting guidelines and the finding that other studies have shown that TCM reporting guidelines do improve the quality of reporting of TCM research [39, 40], it is foreseeable that both the endorsement of reporting guidelines and the quality of TCM research in TCM will improve if the implementation of reporting guidelines continue to be promoted in the future.

Based on the results of regression analysis, whether the inclusion of SCI/SCIE and whether the publication language includes English is associated with the reporting guideline endorsement. On 23, June 2021, an opinion document jointly released by the Central Propaganda Department of the Communist Party of China, Ministry of Education and Ministry of Science and Technology of China proposed to strengthen the bilingual construction of Chinese journals in English and Chinese and improve the academic evaluation system of journals [41]. Combined with our findings, promoting the journals to be published in English and indexed by an international evaluation system like SCI can contribute to the endorsement of reporting guidelines in Chinese medical journals. We believe that those journals only published in Chinese should also endorse reporting guidelines to meet the same standards.

During the past ten years, the efforts of Chinese scholars in promoting the reporting guidelines, especially in the introduction and translation of reporting guidelines, the establishment of the system for TCM reporting guidelines, and the leading role of the ministries of China in promoting the internationalization of journals should be admitted. However, there is a long way to go to be optimal. This current gap is likely to make it difficult to accurately assess the quality of clinical research in China and track the raw data. It will also damage the credibility of Chinese clinical research in the international community. Knowing but not doing it will lead to research waste [41–43]. Some studies suggest that one of the barriers to the implementation of reporting guidelines in Chinese medical journals is the low level of awareness of reporting guidelines among stakeholders such as journal editors [44, 45]. To enhance the use of reporting guidelines in China, we proposed the following initiatives.

First, as the final guarantee to medical research publication, journals should take action to safeguard the reporting quality of medical research, for example, adopting the reporting guidelines as “active strong” [46]. Second, the authors, reviewers, editors, and other stakeholders must work together to ensure that research is reported in line with the relevant reporting guidelines. Third, the 1039 Chinese journal editors should be surveyed to find out their needs regarding implementing reporting guidelines and other issues, including implementing open science practices. Based on their need, the corresponding training courses could be provided. Forth, establishing bases of international reporting guidelines network in China. In January 2021, the Chinese EQUATOR centre launched [47]. The Chinese EQUATOR centre will implement the EQUATOR Network's vision and mission, thus promoting the reporting guidelines in China. Fifth, from the national level, the journals included by the CPA should require the use of reporting guidelines as the Chinese Medical Association (CMA). Our previous study indicated that 69 journals of the CMA used a unified submission system, all of which recommended the use of reporting guidelines [48]. Sixth, we recommend that promoting the Chinese medical journals into the world journal evaluation system, publishing in English, and endorsing the reporting guidelines could be carried out simultaneously.

Our study has limitations. First, we only searched online databases without manual search, thus may omit some medical journals published in print only. Second, there is a certain degree of information delay as the collection of journal information is a one-off and the content of journal websites are updated in real-time. Third, due to the large amount of Chinese medical journals, the researchers did not extract and assess the data independently, although we introduced other researchers to double-check the results, which may cause potential bias in the conclusion. In order to assure the accuracy and transparency of the study results, we provided links to the extracted sources for each record and permanently stored them on the OSF platform for readers to access.

## Conclusions

In conclusion, the endorsement of reporting guidelines in Chinese medical journals remains far from optimal. If the Chinese scientific community wants to improve and safeguard the quality and transparency of medical research, effective implementation strategies must be taken to promote the use of reporting guidelines in China [49, 50, 51].

## Supplementary Information

**Additional file 1:** **Appendix1. **Included Journals.

## Data Availability

The datasets used and/or analysed during the current study are available from the corresponding author on reasonable request. The lead authors (the manuscript’s guarantors) affirm that the manuscript is an honest, accurate, and transparent account of the study being reported; that no important aspects of the study have been omitted; and that any discrepancies from the study as originally planned (and, if relevant, registered) have been explained.
