# Supplementary material for: A cross-sectional study of the endorsement proportion of reporting guidelines in 1039 Chinese medical journals
Source: BMC Med Res Methodol. 2023 Jan 21;23:20. doi: 10.1186/s12874-022-01789-1 (PMC9862842; doi:10.1186/s12874-022-01789-1)
Supplement: Supplementary file 1 — Additional file 1: Appendix1. Included Journals. [file 12874_2022_1789_MOESM1_ESM.docx]

**Appendix 1. Included Journals**

1. Academic Journal of Chinese PLA Medical School
2. Academic Journal of Guangzhou Medical University
3. Academic Journal of Second Military Medical University
4. ACTA ACADEMIAE MEDICINAE SINICAE
5. Acta Anatomica Sinica
6. Acta Cardiologica Sinica
7. Acta Chinese Medicine and Pharmacology
8. Acta Laser Biology Sinica
9. Acta Medicinae Sinica
10. Acta Medicinae Universitatis Scientiae et Technologiae Huazhong
11. Acta Pharmaceutica Sinica B
12. Acta Pharmacologica Sinica
13. Acta Psychologica Sinica
14. Acta Universitatis Medicinalis Anhui
15. Acupuncture Research
16. Adaptive Medicine
17. Advances in Cardiovascular Diseases
18. Adverse Drug Reactions Journal
19. Aerospace Medicine
20. Anhui Journal of Preventive Medicine
21. Anhui Medical and Pharmaceutical Journal
22. ANHUI YIXUE
23. Annals of Nuclear Medicine and Molecular Imaging
24. Anti-Infection Pharmacy
25. Applied Laser
26. Applied Preventive Medicine
27. Archives of Clinical Psychology
28. Asian Journal of Andrology
29. Asian pacific Journal of Reproduction
30. Asian Pacific Journal of Tropical Disease
31. Asian Pacific Journal of Tropical Medicine
32. Asia-Pacific Traditional Medicine
33. BACHU MEDICAL JOURNAL
34. Basic & Clinical Medicine
35. Beijing Journal of Stomatology
36. Beijing Journal of Traditional Chinese Medicine
37. Biomedical Engineering and Clinical Medicine
38. Biotic Resources
39. Bone Research
40. Brain Science Advances
41. Bulletin of Disease Control & Prevention (China)
42. Bulletin of Science and Technology
43. Burns & Trauma
44. Cancer Biology & Medicine
45. Cancer Research and Clinic
46. Cancer Research on Prevention and Treatment
47. Cardiovascular Innovations and Applications
48. Caring for Love Quarterly
49. Cellular & Molecular Immunology
50. Central South Pharmacy
51. Chang Gung Journal of Science
52. Chang Gung Nursing
53. Cheng Ching Medical Journal
54. China & Foreign Medical Treatment
55. China Cancer
56. China Clinical Practical Medicine
57. China Continuing Medical Education
58. China Digital Medicine
59. China Health Industry
60. China Health Standard Management
61. China Journal of Chinese Materia Medica
62. China Journal of Chinese Ophthalmology
63. China Journal of Endoscopy
64. China Journal of Leprosy and Skin Diseases
65. China Journal of Modern Medicine
66. China Journal of Oral and Maxillofacial Surgery
67. China Journal of Orthopaedics and Traumatology
68. China Journal of Pharmaceutical Economics
69. China Journal of Traditional Chinese Medicine and Pharmacy
70. China Medical Device Information
71. China Medical Devices
72. China Medical Engineering
73. China Medical Equipment
74. China Medical Herald
75. China Medical News
76. China Medicine
77. China Medicine and Pharmacy
78. China Modern Doctor
79. China Modern Medicine
80. China Mongolian Medicine Magazine
81. China Oncology
82. China Pharmaceuticals
83. China Pharmacist
84. China Pharmacy
85. China Practical Medicine
86. China Rural Health
87. China Tropical Medicine
88. China's Naturopathy
89. Chinese Acupuncture & Moxibustion
90. Chinese and Foreign Medical Research
91. Chinese Archives of General Surgery(Electronic Edition)
92. Chinese Archives of Otolaryngology-Head and Neck Surgery
93. Chinese Archives of Traditional Chinese Medicine
94. Chinese Baby
95. Chinese Circulation Journal
96. Chinese Clinical Nursing
97. Chinese Clinical Oncology
98. Chinese Community Doctors
99. Chinese Critical Care Medicine
100. Chinese Evidence-Based Nursing
101. Chinese General Practice
102. Chinese General Practice Nursing
103. Chinese Group Psychotherapy
104. Chinese Health Care
105. Chinese Heart Journal
106. Chinese Hepatology
107. Chinese Imaging Journal of Integrated Traditional and Western Medicine
108. Chinese Journal for Clinicians
109. Chinese Journal of Acupuncture and Moxibustion (Electronic Edition)
110. Chinese Journal of Aerospace Medicine
111. Chinese Journal of Aesthetic and Plastic Surgery
112. Chinese Journal of AIDS & STD
113. Chinese Journal of Allergy and Clinical Immunology
114. Chinese Journal of Anatomy and Clinics
115. Chinese Journal of Andrology
116. Chinese Journal of Anesthesiology
117. Chinese Journal of Antituberculosis
118. Chinese Journal of Applied Clinical Pediatrics
119. Chinese Journal of Arteriosclerosis
120. Chinese Journal of Bases and Clinics in General Surgery
121. Chinese Journal of Basic Medicine in Traditional Chinese medicine
122. Chinese Journal of Behavioral Medicine and Brain Science
123. Chinese Journal of Biologicals
124. Chinese Journal of Biomedical Engineering
125. Chinese Journal of Biomedical Engineering
126. Chinese Journal of Blood Purification
127. Chinese Journal of Blood Transfusion
128. Chinese Journal of Bone and Joint
129. Chinese Journal of Bone and Joint Injury
130. Chinese Journal of Bone and Joint Surgery
131. Chinese Journal of Brain Diseases and Rehabilitation (Electronic Edition)
132. Chinese Journal of Breast Disease (Electronic Edition)
133. Chinese Journal of Burns
134. Chinese Journal of Cancer
135. Chinese Journal of Cancer Biotherapy
136. Chinese Journal of Cancer Prevention and Treatment
137. Chinese Journal of Cancer Research
138. Chinese Journal of Cardiac Arrhythmias
139. Chinese Journal of Cardiac Pacing and Electrophysiology
140. Chinese Journal of Cardiology
141. Chinese Journal of Cardiovascular Medicine
142. Chinese Journal of Cardiovascular Research
143. Chinese Journal of Cell and Stem Cell (Electronic Edition)
144. Chinese Journal of Cerebrovascular Diseases
145. Chinese Journal of Cerebrovascular Diseases (Electronic Edition)
146. Chinese Journal of Child Health Care
147. Chinese Journal of Clinical Anatomy
148. Chinese Journal of Clinical Healthcare
149. Chinese Journal of Clinical Infectious Diseases
150. Chinese Journal of Clinical Medicine
151. Chinese Journal of Clinical Neurosurgery
152. Chinese Journal of Clinical Nutrition
153. Chinese Journal of Clinical Obstetrics and Gynecology
154. Chinese Journal of Clinical Oncology
155. Chinese Journal of Clinical Oncology and Rehabilitation
156. Chinese Journal of Clinical Pharmacology and Therapeutics
157. Chinese Journal of Clinical Pharmacy
158. Chinese Journal of Clinical Psychology
159. Chinese Journal of Clinical Rational Drug Use
160. Chinese Journal of Clinical Research
161. Chinese Journal of Clinical Thoracic and Cardiovascular Surgery
162. Chinese Journal of Clinicians (Electronic Edition)
163. Chinese Journal of Coal Industry Medicine
164. Chinese Journal of Colorectal Diseases (Electronic Edition)
165. Chinese Journal of Contemporary Neurology and Neurosurgery
166. Chinese Journal of Contemporary Pediatrics
167. Chinese Journal of Control of Endemic Diseases
168. Chinese Journal of Convalescent Medicine
169. Chinese Journal of Critical Care & Intensive Care Medicine (Electronic Edition)
170. Chinese Journal of Critical Care Medicine
171. Chinese Journal of Critical Care Medicine (Electronic Edition)
172. Chinese Journal of Current Advances in General Surgery
173. Chinese Journal of Dental Materials and Devices
174. Chinese Journal of Dermatology
175. Chinese Journal of Diabetes
176. Chinese Journal of Diabetes Mellitus
177. Chinese Journal of Diagnostics (Electronic Edition)
178. Chinese Journal of Dialysis and Artificial Organs
179. Chinese Journal of Difficult and Complicated Cases
180. Chinese Journal of Digestion
181. Chinese Journal of Digestive Endoscopy
182. Chinese Journal of Digestive Surgery
183. Chinese Journal of Disaster Medicine
184. Chinese Journal of Disease Control & Prevention
185. Chinese Journal of Drug Abuse Prevention and Treatment
186. Chinese Journal of Drug Dependence
187. Chinese Journal of Drug Evaluation
188. Chinese Journal of Emergency and Critical Care Nursing
189. Chinese Journal of Emergency Medicine
190. Chinese Journal of Endemiology
191. Chinese Journal of Endocrine Surgery
192. Chinese Journal of Endocrinology and Metabolism
193. Chinese Journal of Endourology (Electronic Edition)
194. Chinese Journal of Epidemiology
195. Chinese Journal of Ethnomedicine and Ethnopharmacy
196. Chinese Journal of Evidence-Based Cardiovascular Medicine
197. Chinese Journal of Evidence-Based Medicine
198. Chinese Journal of Evidence-Based Pediatrics
199. Chinese Journal of Experimental and Clinical Infectious Diseases(Electronic Edition)
200. Chinese Journal of Experimental and Clinical Virology
201. Chinese Journal of Experimental Ophthalmology
202. Chinese Journal of Experimental Surgery
203. Chinese Journal of Experimental Traditional Medical Formulae
204. Chinese Journal of Extracorporeal Circulation
205. Chinese Journal of Family Planning & Gynecotokology
206. Chinese Journal of Gastroenterology and Hepatology
207. Chinese Journal of Gastroesophageal Reflux Disease (Electronic Edition)
208. Chinese Journal of Gastrointestinal Endoscopy (Electronic Edition)
209. Chinese Journal of Gastrointestinal Surgery
210. Chinese Journal of General Practice
211. Chinese Journal of General Practitioners
212. Chinese Journal of General Surgery
213. Chinese Journal of General Surgery
214. Chinese Journal of Geriatric Dentistry
215. Chinese Journal of Geriatric Heart Brain and Vessel Diseases
216. Chinese Journal of Geriatric Orthopaedics & Rehabilitation(Electronic Edition)
217. Chinese Journal of Geriatrics
218. Chinese Journal of Geriatrics Research (Electronic Edition)
219. Chinese Journal of Gerontology
220. Chinese Journal of Hand Surgery
221. Chinese Journal of Health Care and Medicine
222. Chinese Journal of Health Management
223. Chinese Journal of Heart and Heart Rhythm (Electronic Edition)
224. Chinese Journal of Heart Failure and Cardiomyopathy
225. Chinese Journal of Hematology
226. Chinese Journal of Hepatobiliary Surgery
227. Chinese Journal of Hepatology
228. Chinese Journal of Hernia and Abdominal Wall Surgery (Electronic Edition)
229. Chinese Journal of Hospital Pharmacy
230. Chinese Journal of Human Sexuality
231. Chinese Journal of Hygiene Rescue (Electronic Edition)
232. Chinese Journal of Hypertension
233. Chinese Journal of Industrial Hygiene and Occupational Diseases
234. Chinese Journal of Industrial Medicine
235. Chinese Journal of Infection and Chemotherapy
236. Chinese Journal of Infection Control
237. Chinese Journal of Infectious Diseases
238. Chinese Journal of Inflammatory Bowel Diseases
239. Chinese Journal of Information on Traditional Chinese Medicine
240. Chinese Journal of Injury Repair and Wound Healing (Electronic Edition)
241. Chinese Journal of Integrated Traditional and Western Medicine
242. Chinese Journal of Integrated Traditional and Western Medicine in Intensive and Critical Care
243. Chinese Journal of Integrated Traditional and Western Medicine on Digestion
244. Chinese Journal of Integrative Medicine
245. Chinese Journal of Integrative Medicine on Cardio-Cerebrovascular Disease
246. Chinese Journal of Internal Medicine
247. Chinese Journal of Interventional Cardiology
248. Chinese Journal of Interventional Imaging and Therapy
249. Chinese Journal of Interventional Radiology (Electronic Edition)
250. Chinese Journal of Joint Surgery (Electronic Edition)
251. Chinese Journal of Kidney Disease Investigation(Electronic Edition)
252. Chinese Journal of Laparoscopic Surgery (Electronic Edition)
253. Chinese Journal of Liver Diseases (Electronic Version)
254. Chinese Journal of Lung Cancer
255. Chinese Journal of Lung Diseases (Electronic Edition)
256. Chinese Journal of Marine Drugs
257. Chinese Journal of Medical Aesthetics and Cosmetology
258. Chinese Journal of Medical Genetics
259. Chinese Journal of Medical Instrumentation
260. Chinese Journal of Medical Physics
261. Chinese Journal of Medical Ultrasound (Electronic Edition)
262. Chinese Journal of Medicinal Guide
263. Chinese Journal of Medicine
264. Chinese Journal of Microbiology and Immunology
265. Chinese Journal of Microcirculation
266. Chinese Journal of Microsurgery
267. Chinese Journal of Minimally Invasive Neurosurgery
268. Chinese Journal of Minimally Invasive Surgery
269. Chinese Journal of Misdiagnostics
270. Chinese Journal of Modern Applied Pharmacy
271. Chinese Journal of Modern Applied Pharmacy
272. Chinese Journal of Modern Nursing
273. Chinese Journal of Modern Operative Surgery
274. Chinese Journal of Multiple Organ Disease in the Elderly
275. Chinese Journal of Mycology
276. Chinese Journal of Nautical Medicine and Hyperbaric Medicine
277. Chinese Journal of Neonatology
278. Chinese Journal of Nephrology
279. Chinese Journal of Nephrology, Dialysis & Transplantation
280. Chinese Journal of Nervous and Mental Diseases
281. Chinese Journal of Neuroimmunology and Neurology
282. Chinese Journal of Neurology
283. Chinese Journal of Neuromedicine
284. Chinese Journal of Neurosurgery
285. Chinese Journal of Neurotraumatic Surgery (Electronic Edition)
286. Chinese Journal of New Clinical Medicine
287. Chinese Journal of New Drugs
288. Chinese Journal of New Drugs and Clinical Remedies
289. Chinese Journal of Nuclear Medicine and Molecular Imaging
290. Chinese Journal of Nursing
291. Chinese Journal of Obesity and Metabolic Diseases (Electronic Edition)
292. Chinese Journal of Obstetrics and Gynecology
293. Chinese Journal of Occupational Medicine
294. Chinese Journal of Ocular Fundus Diseases
295. Chinese Journal of ocular trauma and occupational eye disease
296. Chinese Journal of Oncology
297. Chinese Journal of Oncology Prevention and Treatment
298. Chinese Journal of Operative Procedures of General Surgery (Electronic Edition)
299. Chinese Journal of Ophthalmology
300. Chinese Journal of Ophthalmology and Otorhinolaryngology
301. Chinese Journal of Optometry Ophthalmology and Visual Science
302. Chinese Journal of Oral Implantology
303. Chinese Journal of Organ Transplantation
304. Chinese Journal of Orthodontics
305. Chinese Journal of Orthopaedic Trauma
306. Chinese Journal of Orthopaedics
307. Chinese Journal of Osteoporosis
308. Chinese Journal of Osteoporosis and Bone Mineral Research
309. Chinese Journal of Otology
310. Chinese Journal of Otorhinolaryngology Head and Neck Surgery
311. Chinese Journal of Otorhinolaryngology-Skull Base Surgery
312. Chinese Journal of Pain Medicine
313. Chinese Journal of Painology
314. Chinese Journal of Pancreatology
315. Chinese Journal of Parasitology and Parasitic Diseases
316. Chinese Journal of Pathology
317. Chinese Journal of Pediatric Surgery
318. Chinese Journal of Pediatrics
319. Chinese Journal of Perinatal Medicine
320. Chinese Journal Of Pharmaceutical Biotechnology
321. Chinese Journal of Pharmacoepidemiology
322. Chinese Journal of Pharmacovigilance
323. Chinese Journal of Physical Medicine and Rehabilitation
324. Chinese Journal of Plastic and Reconstructive Surgery
325. Chinese Journal of Plastic Surgery
326. Chinese Journal of Postgraduates of Medicine
327. Chinese Journal of Practical Gynecology and Obstetrics
328. Chinese Journal of Practical Internal Medicine
329. Chinese Journal of Practical Medicine
330. Chinese Journal of Practical Nervous Diseases
331. Chinese Journal of Practical Nursing
332. Chinese Journal of Practical Ophthalmology
333. Chinese Journal of Practical Pediatrics
334. Chinese Journal of Practical Stomatology
335. Chinese Journal of Practical Surgery
336. Chinese Journal of Preventive Medicine
337. Chinese Journal of Primary Medicine and Pharmacy
338. Chinese Journal of Prosthodontics
339. Chinese Journal of Psychiatry
340. Chinese Journal of Radiological Health
341. Chinese Journal of Radiological Medicine and Protection
342. Chinese Journal of Radiology
343. Chinese Journal of Rational Drug Use
344. Chinese Journal of Rehabilitation Medicine
345. Chinese Journal of Rehabilitation Theory and Practice
346. Chinese Journal of Reparative and Reconstructive Surgery
347. Chinese Journal of Reproduction and Contraception
348. Chinese Journal of Reproductive Health
349. Chinese Journal of Respiratory and Critical Care Medicine
350. Chinese Journal of Rheumatology
351. Chinese Journal of Robotic Surgery
352. Chinese Journal of Rural Medicine and Pharmacy
353. Chinese Journal of Schistosomiasis Control
354. Chinese Journal of School Doctor
355. Chinese Journal of Shoulder and Elbow(Electronic Edition)
356. Chinese Journal of Spine and Spinal Cord
357. Chinese Journal of Sports Medicine
358. Chinese Journal of Stereotactic and Functional Neurosurgery
359. Chinese Journal of Stomatological Continuing Education
360. Chinese Journal of Stomatological Research (Electronic Edition)
361. Chinese Journal of Stomatology
362. Chinese Journal of Stroke
363. Chinese Journal of Surgery
364. Chinese Journal of Surgery of Integrated Traditional and Western Medicine
365. Chinese Journal of the Frontiers of Medical Science (Electronic Version)
366. Chinese Journal of Thoracic and Cardiovascular Surgery
367. Chinese Journal of Thoracic Surgery (Electronic Edition)
368. Chinese Journal of Tissue Engineering Research
369. Chinese Journal of Traditional Medical Traumatology & Orthopedics
370. Chinese Journal of Trauma
371. Chinese Journal of Trauma and Disability Medicine
372. Chinese Journal of Traumatology
373. Chinese Journal of Tuberculosis and Respiratory Diseases
374. Chinese Journal of Ultrasonography
375. Chinese Journal of Ultrasound in Medicine
376. Chinese Journal of Urban and Rural Enterprise Hygiene
377. Chinese Journal of Urology
378. Chinese Journal of Vascular Surgery
379. Chinese Journal of Vascular Surgery (Electronic Version)
380. Chinese Journal of Viral Diseases
381. Chinese Journal of Woman and Child Health Research
382. Chinese Journal of Women and Children Health
383. Chinese Manipulation and Rehabilitation Medicine
384. Chinese Medical Equipment Journal
385. Chinese Medical Journal
386. Chinese Medical Journal of Metallurgical Industry
387. Chinese Medical Sciences Journal
388. Chinese Medicinal Biotechnology
389. Chinese Medicine Modern Distance Education of China
390. Chinese Nursing Management
391. Chinese Orthopaedic Journal of Clinical and Basic Research
392. Chinese Pediatric Emergency Medicine
393. Chinese Pediatrics of Integrated Traditional and Western Medicine
394. Chinese Pharmaceutical Affairs
395. Chinese Pharmaceutical Journal
396. Chinese Practical Journal of Rural Doctor
397. Chinese Remedies & Clinics
398. Chinese Scientific Journal of Hearing and Speech Rehabilitation
399. Chinese Traditional and Herbal Drugs
400. CHINESE TRADITIONAL PATENT MEDICINE
401. Chinse Nursing Research
402. Chongqing Medicine
403. Chronic Diseases and Translational Medicine
404. Chronic Pathematology Journal
405. Classical Chinese Medicine Research
406. Clinical Education of General Practice
407. Clinical Focus
408. Clinical Journal of Chinese Medicine
409. Clinical Journal of Medical Officers
410. Clinical Journal of Traditional Chinese Medicine
411. Clinical Medication Journal
412. Clinical Medicine & Engineering
413. Clinical Medicine of China
414. Clinical Misdiagnosis & Mistherapy
415. Clinical Research
416. Clinical Research and Practice
417. Contemporary Medicine
418. Continuing Medical Education
419. Current Immunology
420. Current Medical Science
421. Diabetes New World
422. Diagnostic Imaging & Interventional Radiology
423. Diet Health
424. Digital Chinese Medicine
425. Doctor
426. Doctor Online Magazine
427. Drug Combination Therapy
428. Drug Evaluation
429. Drug Evaluation Research
430. Drugs & Clinic
431. Editorial Board of Acta Academiae Medicinae Wannan
432. Editorial Board of Journal of Medical Science Yanbian University
433. Electronic Journal of Emerging Infectious Diseases
434. Electronic Journal of Liver Tumor
435. Electronic Journal of Metabolism and Nutrition of Cancer
436. Electronic Journal of Practical Clinical Nursing Science
437. Evaluation and Analysis of Drug-Use in Hospitals of China
438. Experimental and Laboratory Medicine
439. Eye and Vision
440. Eye Science
441. Food and Drug
442. Food Therapy and Health Care
443. Formosan Journal of Endocrinology and Metabolism
444. Formosan Journal of Medicine
445. Formosan Journal of Physical Therapy
446. Forum on Traditional Chinese Medicine
447. Frontiers of Medicine
448. Frontiers of Nursing
449. Fudan University Journal of Medical Sciences
450. Fu-Jen Journal of Medicine
451. Fujian Medical Journal
452. Gansu Medical Journal
453. General Psychiatry
454. Genomics and Applied Biology
455. Geriatrics & Health Care
456. Global Traditional Chinese Medicine
457. Guangdong Medical Journal
458. Guangming Journal of Chinese Medicine
459. Guangxi Journal of Traditional Chinese Medicine
460. Guangxi Medical Journal
461. Guangzhou Medical Journal
462. Guide of China Medicine
463. Guiding Journal of Traditional Chinese Medicine and Pharmacy
464. Guizhou Medical Journal
465. Hainan Medical Journal
466. Harbin Medical Journal
467. Health Education and Health Promotion
468. Health for Everyone
469. Health Guide
470. Health Medicine Research and Practice
471. Health Promotion Research and Practice
472. Health Research
473. Health Vocational Education
474. Hebei Journal of Traditional Chinese Medicine
475. Hebei Medical Journal
476. Hebei Medicine
477. Heilongjiang Medical Journal
478. Henan Journal of Preventive Medicine
479. Henan Journal of Surgery
480. Henan Medical Research
481. Henan Traditional Chinese Medicine
482. Hepatobiliary & Pancreatic Diseases International
483. Hepatobiliary Surgery and Nutrition
484. Hepatoma Research
485. Herald of Medicine
486. Hong Kong Medical Journal
487. Hospital
488. Hospital Management Forum
489. Hubei Journal of Traditional Chinese Medicine
490. Hunan Journal of Traditional Chinese Medicine
491. Industrial Health and Occupational Diseases
492. Infection, Inflammation, Repair
493. Infectious Disease Information
494. Infectious Diseases of Poverty
495. Information on Traditional Chinese Medicine
496. Inner Mongolia Journal of Traditional Chinese Medicine
497. Inner Mongolia Medical Journal
498. Internal Medicine
499. International Eye Science
500. International Journal of Anesthesiology and Resuscitation
501. International Journal of Biomedical Engineering
502. International Journal of Blood Transfusion and Hematology
503. International Journal of Cardiovascular Disease
504. International Journal of Cardiovascular Diseases
505. International Journal of Cerebrovascular Diseases
506. International Journal of Dermatology And Venereology
507. International Journal of Epidemiology and Infectious Disease
508. International Journal of Geriatrics
509. International Journal of Gerontology
510. International Journal of Immunology
511. International Journal of Laboratory Medicine
512. International Journal of Nursing
513. International Journal of Nursing Sciences
514. International Journal of Ophthalmology
515. International Journal of Oral Science
516. International Journal of Orthopaedics
517. International Journal of Pediatrics
518. International Journal of Radiation Medicine and Nuclear Medicine
519. International Journal of Respiration
520. International Journal of Stomatology
521. International Journal of Surgery
522. International Journal of TCM Aromatherapy
523. International Journal of Traditional Chinese Medicine
524. International Journal of Transplantation and Hemopurification
525. International Journal of Urology and Nephrology
526. International Journal of Virology
527. JiaJi Nursing
528. JIAN KANG QIAN YAN
529. Jiangsu Journal of Traditional Chinese Medicine
530. Jiangsu Medical Journal
531. Jiangxi Journal of Traditional Chinese Medicine
532. Jiangxi Medical Journal
533. Jilin Journal of Chinese Medicine
534. Jilin Medical Journal
535. Journal of Abdominal Surgery
536. Journal of Acupuncture and Tuina Science
537. Journal of Acute Disease
538. Journal of Acute Medicine
539. Journal of Anhui Health Vocational & Technical College
540. Journal of Anhui University of Chinese Medicine
541. Journal of Apoplexy and Nervous Diseases
542. Journal of Audiology and Speech Pathology
543. Journal of Baotou Medical College
544. JOURNAL OF BEIHUA UNIVERSITY (NATURAL SCIENCE)
545. Journal of Beijing University od Traditional Chinese Medicine
546. Journal of Binzhou Medical University
547. Journal of Bio-education
548. Journal of Brain and Nervous Diseases
549. Journal of Campus Life & Mental Health
550. Journal of Cancer Control and Treatment
551. Journal of Cancer Metastasis and Treatment
552. Journal of Capital Medical University
553. Journal of Cardiovascular and Pulmonary Diseases
554. Journal of Central South University (Medical Science)
555. Journal of Changchun University of Chinese Medicine
556. Journal of Changzhi Medical College
557. Journal of Chengde Medical College
558. Journal of Chengdu Medical College
559. Journal of Chengdu University of Traditional Chinese Medicine
560. Journal of China Clinic Medical Imaging
561. Journal of China Medical University
562. Journal of China Pediatric Blood and Cancer
563. Journal of China Prescription Drug
564. Journal of China-Japan Friendship Hospital
565. Journal of Chinese Medical Acupuncture Science
566. Journal of Chinese Medicine
567. Journal of Chinese Medicine
568. Journal of Chinese Oncology
569. Journal of Chinese Ophthalmology and Otorhinolaryngology
570. Journal of Chinese Pharmaceutical Sciences
571. Journal of Chinese Physician
572. Journal of Chinese Practical Diagnosis and Therapy
573. Journal of Chinese Research Hospitals
574. Journal of Chongqing Medical University
575. Journal of Clinical Acupuncture and Moxibustion
576. Journal of Clinical and Experimental Medicine
577. Journal of Clinical and Pathological Research
578. Journal of Clinical and Translational Hepatology
579. Journal of Clinical Cardiology
580. Journal of Clinical Dermatology
581. Journal of Clinical Emergency
582. Journal of Clinical Hematology
583. Journal of Clinical Hematology (Blood Transfusion & Laboratory Medicine)
584. Journal of Clinical Hepatology
585. Journal of Clinical Medicine in Practice
586. Journal of Clinical Nephrology
587. Journal of Clinical Neurology
588. Journal of Clinical Nursing
589. Journal of Clinical Ophthalmology
590. Journal of Clinical Orthopaedics
591. Journal of Clinical Orthopedics and Research
592. Journal of Clinical Otorhinolaryngology Head and Neck Surgery
593. Journal of Clinical Pediatric Surgery
594. Journal of Clinical Pediatrics
595. Journal of Clinical Pulmonary Medicine
596. Journal of Clinical Radiology
597. Journal of Clinical Research
598. Journal of Clinical Stomatology
599. Journal of Clinical Surgery
600. Journal of Clinical Transfusion and Laboratory Medicine
601. Journal of Clinical Ultrasound in Medicine
602. Journal of Clinical Urology
603. Journal of Colorectal & Anal Surgery
604. Journal of Community Medicine
605. Journal of Contemporary Clinical Medicine
606. Journal of Dali University
607. Journal of Dalian Medical University
608. Journal of Dermatology and Venereology
609. Journal of Developmental Medicine (Electronic Version)
610. Journal of Diagnosis and Therapy on Dermato-venereology
611. Journal of Digestive Oncology (Electronic Version)
612. Journal of Diseases Monitor & Control
613. Journal of Electrocardiology and Circulation
614. Journal of Emergency in Traditional Chinese Medicine
615. Journal of Enhanced Recovery After Surgery
616. Journal of Epilepsy
617. Journal of Esophageal Diseases
618. Journal of Evidence-Based Medicine
619. Journal of Exercise Physiology and Fitness
620. Journal of Experimental Hematology
621. Journal of External Therapy of Traditional Chinese Medicine
622. Journal of Fujian Medical University
623. Journal of Fujian University of Traditional Chinese Medicine
624. Journal of Gannan Medical University
625. Journal of Gansu University of Chinese Medicine
626. Journal of Geriatric Cardiology
627. Journal of Guangdong Medical University
628. Journal of Guangdong Pharmaceutical University
629. Journal of Guangxi Medical University
630. Journal of Guangxi University of Chinese Medicine
631. Journal of Guangzhou University of Traditional Chinese Medicine
632. Journal of Guizhou Medical University
633. Journal of GuiZhou University of Traditional Chinese Medicine
634. Journal of Hainan Medical College
635. Journal of Harbin Medical University
636. Journal of Health and Architecture
637. Journal of Hebei Medical University
638. Journal of Henan Medical College
639. Journal of Henan University (Medical Science)
640. Journal of Hepatobiliary Surgery
641. Journal of Hepatopancreatobiliary Surgery
642. Journal of Heze Medical College
643. Journal of High Altitude Medicine
644. Journal of Huaihai Medicine
645. Journal Of HuBei Minzu University (Medical Edition)
646. Journal of Hubei University of Chinese Medicine
647. Journal of Hubei University of Medicine
648. Journal of Hubei University of Science and Technology (Medical Sciences)
649. Journal of Hunan Normal University (Medical Sciences)
650. Journal of Hunan University of Chinese Medicine
651. Journal of Imaging Research and Medical Applications
652. Journal of Inner Mongolia Medical University
653. Journal of Integrated Chinese and Western Medicine
654. Journal of Integrative Nursing
655. Journal of Internal Intensive Medicine
656. Journal of Internal Medicine Concepts & Practice
657. Journal of International Neurology and Neurosurgery
658. Journal of International Obstetrics and Gynecology
659. Journal of International Oncology
660. Journal of International Psychiatry
661. Journal of International Reproductive Health
662. Journal of International Translational Medicine
663. Journal of Interventional Medicine
664. Journal of Interventional Radiology
665. Journal of Jianghan University (Natural Science Edition)
666. Journal of Jiangxi University of Traditional Chinese Medicine
667. Journal of Jilin University (Medicine Edition)
668. Journal of Jinan University(Natural Science & Medicine Edition)
669. Journal of Jining Medical University
670. Journal of Jiujiang University (Natural Science Edition)
671. Journal of Kunming Medical University
672. Journal of Lanzhou University (Medical Sciences)
673. Journal of Laparoscopic Surgery
674. Journal of Leukemia and Lymphoma
675. Journal of Liaoning University of Traditional Chinese Medicine
676. Journal of Logistics University of PAP (Medical Sciences)
677. Journal of Mathematical Medicine
678. Journal of Medical Aesthetics and Cosmetology
679. Journal of Medical and Biological Engineering
680. Journal of Medical Imaging
681. Journal of Medical Information
682. Journal of Medical Pest Control
683. Journal of Medical Postgraduates
684. Journal of Medical Research
685. Journal of Medical Sciences
686. Journal of Medicine & Pharmacy of Chinese Minorities
687. Journal of Medicine and health
688. Journal of Military Surgeon in Southwest China
689. Journal of Minimally Invasive Medicine
690. Journal of Minimally Invasive Urology
691. Journal of Modern Clinical Medicine
692. Journal of Modern Medicine & Health
693. Journal of Modern Oncology
694. Journal of Molecular Diagnostics and Therapy
695. Journal of Molecular Imaging
696. Journal of Mudanjiang Medical University
697. Journal of Multidisciplinary Cancer Management (Electronic Version)
698. Journal of Nanchang University (Medical Sciences)
699. Journal of Nanjing Medical University (Natural Sciences)
700. Journal of Nanjing University of Traditional Chinese Medicine
701. Journal of Nanjing University of Traditional Chinese Medicine (Social Science Edition)
702. Journal of Navy Medicine
703. Journal of Neurology and Neurorehabilitation
704. Journal of Neuroscience and Mental Health
705. Journal of New Chinese Medicine
706. Journal of New Medicine
707. Journal of Nongken Medicine
708. Journal of North China University of Science and Technology (Health Sciences Edition)
709. Journal of North Pharmacy
710. Journal of North Sichuan Medical College
711. Journal of Nurses Training
712. Journal of Nursing (China)
713. Journal of Nursing Administration
714. Journal of Nursing and Rehabilitation
715. Journal of Nursing Science
716. Journal of Nutritional Oncology
717. Journal of Occupational Therapy Association R.O.C.
718. Journal of Oral and Maxillofacial Surgery
719. Journal of Oral Science Research
720. Journal of Otolaryngology and Ophthalmology of Shandong University
721. Journal of Otology
722. Journal of Pancreatology
723. Journal of Pathogen Biology
724. Journal of Pediatric Pharmacy
725. Journal of Pediatrics of Traditional Chinese Medicine
726. Journal of Peking University (Health Sciences)
727. Journal of Pharmaceutical Practice
728. Journal of Pharmaceutical Practice
729. Journal of Pharmaceutical Research
730. Journal of Practical Dermatology
731. Journal of Practical Diabetology
732. Journal of Practical Electrocardiology
733. Journal of Practical Hand Surgery
734. Journal of Practical Hepatology
735. Journal of Practical Medical Techniques
736. Journal of Practical Obstetrics and Gynecology
737. Journal of Practical Oncology
738. Journal of Practical Orthopaedics
739. Journal of Practical Radiology
740. Journal of Practical Shock
741. Journal of Practical Stomatology
742. Journal of Practical Traditional Chinese Internal Medicine
743. Journal of Precision Medicine
744. Journal of Prevention and Treatment for Stomatological Diseases
745. Journal of Preventive Medicine of Chinese People's Liberation Army
746. Journal of Psychiatry
747. Journal of Psychiatry
748. Journal of Qilu Nursing
749. Journal of Qingdao University (Medical Sciences)
750. Journal of Qiqihar Medical University
751. Journal of Rare and Uncommon Diseases
752. Journal of Regional Anatomy and Operative Surgery
753. Journal of Reproductive Medicine
754. Journal of Respiratory Therapy
755. Journal of school health care
756. Journal of Shaanxi University of Chinese Medicine
757. Journal of Shandong University (Health Sciences)
758. Journal of Shandong University of Traditional Chinese Medicine
759. Journal of Shanghai Jiaotong University (Medical Science)
760. Journal of Shanghai University of Traditional Chinese Medicine
761. Journal of Shantou University Medical College
762. Journal of Shanxi Medical University
763. Journal of Shanxi University of Chinese Medicine
764. Journal of Shenyang Medical College
765. Journal of Sichuan of Traditional Chinese Medicine
766. Journal of Sichuan University (Medical Science Edition)
767. Journal of Snake
768. Journal of Society of Colon and Rectal Surgeons，Taiwan
769. Journal of Southeast University (Medical Science Edition)
770. Journal of Southern Medical University
771. Journal of Southwest Medical University
772. Journal of Spinal Surgery
773. Journal of Sun Yat-sun University (Medical Sciences)
774. Journal of Surgery Concepts & Practice
775. Journal of Taipei Associationa of Radiological Technologists
776. Journal of Taishan Medical College
777. Journal of Taiwan Art Therapy
778. Journal of Taiwan Medical Radiation Technology
779. Journal of Taiwan Nephrology Nurses Association
780. Journal of Taiwan Nurse Practitioners
781. Journal of Taiwan Occupational Therapy Research and Practice
782. Journal of Taiwan play Theray
783. Journal of the Chinese Medical Association
784. Journal of the Speech-Language-Hearing Association of Taiwan
785. Journal of The Taiwan Academy of Periodontology
786. Journal of Third Military Medical University
787. Journal of Tianjin Medical University
788. Journal of Tianjin University of Traditional Chinese Medicine
789. Journal of Tissue Engineering and Reconstructive Surgery
790. Journal of Tongji University (Medical Science)
791. Journal of Traditional and Complementary Medicine
792. Journal of Traditional Chinese Internal Medicine
793. Journal of Traditional Chinese Medical Sciences
794. Journal of Traditional Chinese Medicine
795. Journal of Traditional Chinese Medicine
796. Journal of Translational Neuroscience
797. Journal of Traumatic Surgery
798. Journal of Tropical Diseases and Parasitology
799. Journal of Tropical Medicine
800. Journal of Tuberculosis and Lung Disease
801. Journal of Vascular and Endovascular Surgery
802. Journal of Wenzhou Medical University
803. Journal of Women's Association of Endometriosis for the Republic of China
804. Journal of Xi’an Jiaotong University (Medical Sciences)
805. Journal of Xiangnan University (Medical Sciences)
806. Journal of Xinjiang Medical University
807. Journal of Xinxiang Medical University
808. Journal of Xuzhou Medical University
809. Journal of Ya’an University (Medical Science)
810. Journal of Yangtze University (Natural Science Edition)
811. Journal of Youjiang Medical University for Nationalities
812. Journal of Yuanpei University of Science and Technology
813. Journal of Yunnan University of Traditional Chinese Medicine
814. Journal of Zhejiang University (Medical Sciences)
815. Journal of Zhengzhou University (Medical Sciences)
816. Journal of Zunyi Medical University
817. Labeled Immunoassays and Clinical Medicine
818. Laboratory Medicine and Clinic
819. Laser Journal
820. Liaoning Journal of Traditional Chinese Medicine
821. Life Research
822. LINGCHUANG MAZUIXUE ZAZHI
823. Lingnan Modern Clinics in Surgery
824. Macau Journal of Nursing
825. Maternal and Child Health Care of China
826. Maternal-Fetal Medicine
827. Medical & Pharmaceutical Journal of Chinese People’s Liberation Army
828. Medical Diet and Health
829. Medical Equipment
830. Medical Innovation of China
831. Medical Journal of Air Force
832. Medical Journal of Chinese People’s Liberation Army
833. Medical Journal of Chinese People's Health
834. Medical Journal of Kiang Wu
835. Medical Journal of Liaoning
836. Medical Journal of the Chinese People's Armed Police Force
837. Medical Journal of West China
838. Medical Journal of Wuhan University
839. Medical Recapitulate
840. Medical Research and Education
841. Medical Science Journal of Central South China
842. Medica-World Science and Technology
843. Military Medical Journal of South China
844. Military Medical Journal of Southeast China
845. Military Medical Research
846. Military Medical Sciences
847. Modern Chinese Clinical Medicine
848. Modern Clinical Nursing
849. Modern Diagnosis and Treatment
850. Modern Digestion & Intervention
851. Modern Hospitals
852. Modern Instruments & Medical Treatment
853. Modern Journal of Integrated Traditional Chinese and Western Medicine
854. Modern Medical Imageology
855. Modern Medical Journal
856. Modern Medicine Journal of China
857. Modern Nurse
858. Modern Preventive Medicine
859. Modern Traditional Chinese Medicine
860. National Journal of Andrology
861. National Medical Journal of China
862. Negative
863. Neural Injury and Functional Reconstruction
864. Neuroscience Bulletin
865. New Medicine
866. Ningxia Medical Journal
867. Northwest Pharmaceutical Journal
868. Nursing of Integrated Traditional Chinese and Western Medicine
869. Nursing Practice and Research
870. Nutrirional Sciences Journal
871. Occupational Health and Damage
872. Occupational Health and Emergency Rescue
873. Oncology and Translational Medicine
874. Oncology Progress
875. Oncoradiology
876. Ophthalmology in China
877. Oral Biomedicine
878. Organ Transplantation
879. Orthopaedic Biomechanics Materials and Clinical Study
880. Orthopaedics Journal
881. Orthopedic Journal of China
882. Otorhinolaryngology Research in Traditional Chinese Medicine
883. Our Health
884. Parenteral & Enteral Nutrition
885. Pediatric Investigation
886. Pediatrics (Electronic Edition)
887. People's Military Surgeon
888. Periodicals Department of Shenyang Pharmaceutical University
889. Pharmaceutical and Clinical Research
890. Pharmaceutical Care and Research
891. Pharmacology and Clinics of Chinese Materia Medica
892. Pharmacy Today
893. Plastic and Aesthetic Research
894. Practical Clinical Journal of Integrated Traditional Chinese and Western Medicine
895. Practical Clinical Medicine
896. Practical Geriatrics
897. Practical Journal of Cardiac Cerebral Pneumal and Vascular Disease
898. Practical Journal of Clinical Medicine
899. Practical Journal of Medicine & Pharmacy
900. Practical Oncology Journal
901. Practical Pharmacy and Clinical Remedies
902. Practical Preventive Medicine
903. Prasitoses and Infectious Diseases
904. Prevention and Treatment of Cardio-Cerebral-Vascular Disease
905. Prevention and Treatment of Cardiovascular Disease
906. Preventive Medicine
907. Proceeding of Clinical Medicine
908. Progress in Biomedical Engineering
909. Progress in Microbiology and Immunology
910. Progress in Modern Biomedicine
911. Progress in Obstetrics and Gynecology
912. Progress in Pharmaceutical Sciences
913. PSY
914. Psychosomatic Medicine Research
915. Publishing House of Chinese Journal of Family Planning
916. Qingdao Medical Journal
917. Qinghai Medical Journal
918. Recent Advances in Ophthalmology
919. Reflexology and Rehabilitation Medicine
920. Rehabilitation Medicine
921. Reproductive and Developmental Medicine
922. Research and Practice on Chinese Medicine
923. Research of Integrated Traditional Chinese and Western Medicine
924. Rheumatism and Arthritis
925. SHAANXI ZHONGYI
926. Shananxi Medical Journal
927. Shandong Journal of Traditional Chinese Medicine
928. Shandong Medical Journal
929. Shanghai Journal of Acupuncture and Moxibustion
930. Shanghai Journal of Stomatology
931. Shanghai Journal of Traditional Chinese Medicine
932. Shanghai Medical & Pharmaceutical Journal
933. Shanghai Medical Journal
934. Shanghai Nursing
935. Shanxi Medical Journal
936. Shenzhen Journal of Integrated Traditional Chinese and Western Medicine
937. SHIZHENGUOYIGUOYAO
938. Sichuan Mental Health
939. SICHUAN YIXUE
940. Smart Healthcare
941. South China Journal of Cardiovascular Diseases
942. St. Joseph's Hospital Medical & Nursing Journal
943. Stomatology
944. Strait Circulation Journal
945. Strait Journal of Preventive Medicine
946. Strait Pharmaceutical Journal
947. Stroke and Nervous Diseases
948. Studies of Trace Elements and Health
949. Surgical Research and New Technique
950. Systems Medicine
951. Taipei City Medical Journal
952. Taiwan Association of Asthma Education
953. Taiwan Geriatrics & Gerontology
954. Taiwan Journal of Chinese Medicine
955. Taiwan Journal of Dietetics
956. Taiwan Journal of Family Medicine
957. Taiwan Journal of Hospice Palliative Care
958. Taiwan Journal of Oral and Maxillofacial Surgery
959. Taiwan Journal of Pediatric Dentistry
960. Taiwan Journal of Physical Medicine and Rehabilitation
961. Taiwan Journal of Public Health
962. Taiwanese Association of Diabetes Educators Bulletin
963. Taiwanese Journal of Applied Radiation and Lsotopes
964. Taiwanese Journal of Orthodontics
965. Taiwanese Journal of Psychiatry
966. The British Medical Journal (Chinese Edition)
967. The Chinese Journal of Clinical Pharmacology
968. The Chinese Journal of Dermatovenereology
969. The Journal of Biomedical Research
970. The Journal of Cervicodynia and Lumbodynia
971. The Journal of Health Sciences
972. The Journal of Medical Theory and Practice
973. The Journal of Nursing
974. The Journal of Nursing Research
975. The journal of Practical Medicine
976. The Journal of Taiwan Association for Medical Informatics
977. The Journal of Traditional Chinese Orthopedics and Traumatology
978. The Journal of Traditional Medicine
979. The Primary Medical Forum
980. The Taiwan Journal of Pain
981. Thoracic Medicine
982. Tianjin Journal of Nursing
983. Tianjin Journal of Traditional Chinese Medicine
984. Tibetan Medicine
985. TMR Cancer
986. TMR Clinical Research
987. TMR Integrative Medicine
988. TMR Integrative Nursing
989. TMR Modern Herbal Medicine
990. TMR Non-Drug Therapy
991. TMR Theory and Hypothesis
992. Today Nurse (General Edition)
993. Today Nurse (Specialist Edition)
994. Traditional Chinese Drug Research and Clinical Pharmacology
995. Traditional Chinese Medicinal Research
996. Traditional Chinese Medicine Journal
997. Traditional Medicine Research
998. Translational Medicine Journal
999. Translational Neurodegeneration
1000. Trauma and Critical Care Medicine
1001. Tumor
1002. Tungs‘ Medical Journal
1003. Uygur Medicine
1004. VGH Nursing
1005. West China Journal of Pharmaceutical Sciences
1006. West China Journal of Stomatology
1007. West China Medical Journal
1008. Western Journal of Traditional Chinese Medicine
1009. WO HE BAO BEI
1010. Women's Health Research
1011. World Chinese Medicine
1012. World Clinical Drugs
1013. World Journal of Acupuncture-Moxibustion
1014. World Journal of Complex Medicine
1015. World Journal of Emergency Medicine
1016. World Journal of Integrated Traditional and Western Medicine
1017. World Journal of Otorhinolaryngology -Head and Neck Surgery
1018. World Journal of Pediatrics
1019. World Journal of Sleep Medicine
1020. World Journal of Traditional Chinese Medicine
1021. World Latest Medicine
1022. World Notes on Antibiotics
1023. XIANDAI YIXUE YU JIANKANG YANJIU
1024. Xinjiang Medical Journal
1025. YIYAO LUNTAN ZAZHI
1026. YIYAOJIE
1027. Youjiang Medical Journal
1028. Yuan-Yuan Nursing
1029. Yunnan Journal of Traditional Chinese Medicine and Materia Medica
1030. Yunnan MEDICINE JOURNAL
1031. Zhejiang Chinese Medical University XUEBAO
1032. Zhejiang Clinical Medical Journal
1033. Zhejiang Journal of Integrated Traditional Chinese and Western Medicine
1034. Zhejiang Journal of Traditional Chinese Medicine
1035. Zhejiang Journal of Traumatic Surgery
1036. Zhejiang Medicine
1037. Zhong Guo Kang Fu
1038. ZHONGGUO SHAOSHANG CHUANGYANG ZAZHI
1039. ZHONGGUO YILIAO MEIRONG
